# Characterization of thiobarbituric acid derivatives as inhibitors of hepatitis C virus NS5B polymerase

**DOI:** 10.1186/1743-422X-8-18

**Published:** 2011-01-14

**Authors:** Jong-Ho Lee, Sangyoon Lee, Mi Young Park, Heejoon Myung

**Affiliations:** 1 Department of Bioscience and Biotechnology, Hankuk University of Foreign Studies, Yong-In, Gyung-Gi Do 449-791, Korea

## Abstract

In an effort to find chemicals inhibiting the enzymatic activity of the hepatitis C virus (HCV) NS5B polymerase, a series of thiobarbituric acid derivatives were selected from a library provided by Korea Research Institute of Chemical Technology and characterized. The selected compounds exhibited IC_50 _values ranging from 1.7 to 3.8 μM, and EC_50 _values ranging from 12.3 to 20.7 μM against NS5B polymerase of type 1b strain. They showed little effect against type 2a polymerase. One of the compounds, G05, was selected and further characterized. It inhibited the synthesis of RNA by recombinant HCV NS5B polymerase in a dose dependent manner. The CC_50 _value was 77 μM. The inhibition was in a noncompetitive manner with the substrate UTP. The compound did not inhibit the elongation step of RNA synthesis in a single-cycle processive polymerization assay. It inhibited the binding of NS5B polymerase to the template RNA in a dose-dependent manner.

## Findings

The hepatitis C virus causes chronic hepatitis in human, and an estimated 170 million people are infected worldwide [1,2]. However, no vaccine has yet been successful, and no specific inhibitor is currently available other than interferon alpha and ribavirin, where the response rate is lower than 50% and side effects have been reported [3,4].

Nonstructural protein 5B is responsible for HCV genomic replication [5,6], which made it a major target for the development of an antiviral therapy and many compounds have been reported to inhibit this target. Non-nucleoside inhibitors (NNIs) bind to an allosteric site and cause a change in the conformation of the active site in the enzyme, thereby inhibiting the initiation step, whereas pyrophosphate mimics bind to catalytic metal ions in the active site of the protein, thereby inhibiting enzymatic activity. Many NNIs have already been reported. One example is benzimidazoles, which bind to the thumb domain of NS5B [3,7-10], while another is thiophene derivatives which are reversible allosteric inhibitors that also bind to the thumb domain [11], yet the binding sites in the thumb domain for the two inhibitors are different. X ray crystallographic studies have revealed that phenylalanine and dihydropyranone scaffold inhibitors bind to the same site in NS5B, although they have different chemical structures [12,13]. Benzothiadiazine scaffold inhibitors are also known to inhibit the initiation step of RNA synthesis [14,15], yet the binding site and inhibition mechanism are believed to be different from others [16].

While screening a chemical library provided by Korea Research Institute of Chemical Technology, several thiobarbituric acid derivatives were found by the current authors to have inhibitory effects on the HCV NS5B polymerase. This study reports on the characterization of inhibitory mechanism by the compounds.

**Figure 1:**
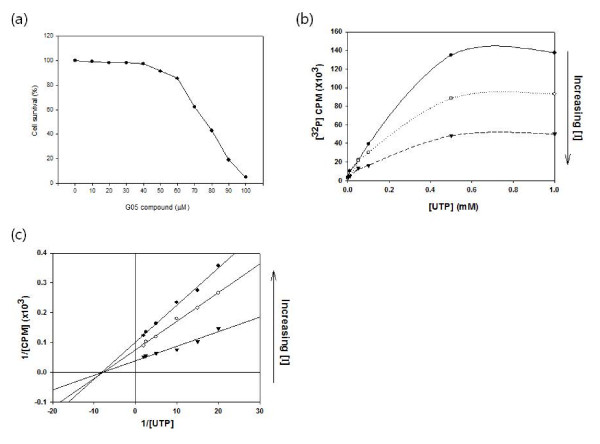
**Mode of inhibition by G05 compound**. (a) Naïve Huh7 cells were treated with various concentrations (up to 100 μM) of G05 compound and the viability was measured with standard MTT assay. (b) Huh7 cells harboring the HCV subgenomic replicon were treated with G05 compound at a concentration of 5 15 or 30 μM. After 72 hours of incubation the amounts of (+) and (-) sense replicon RNA were measured using a real-time RT-PCR. (b) [^32^P]-UMP incorporation measured after 90 minutes of incubation in the presence of G05 at 0 1.7 and 13.5 μM in concentration. (c) The same assay with G05 at 0.1 0.25 and 0.5 μM in concentration and displayed as a Lineweaver-Burk plot. [I] concentration of G05 compound.

6,500 compounds with representative chemical structures from the Korea Research Institute of Chemical Technology (KRICT) were screened for their inhibitory effect on the HCV NS5B polymerase. A bacterial cell-based assay was used for screening as described [17]. The structures of the hit compounds are shown in Additional file 1. All 4 compounds were thiobarbituric acid derivatives. The inhibition of RNA synthesis by these compounds was biochemically tested in a [^32^P]-UMP incorporation assay with a purified recombinant NS5B and poly(A)-oligo(dT) template. Potent inhibition against 1b type polymerase (Con-1) was exhibited with IC_50 _values between 1.7 and 3.8 μM. But essentially no inhibition was observed against 2a (JFH-1) type polymerase. The inhibitory effects on the 1b type HCV subgenomic RNA replicon [18] was measured using a real-time RT-PCR analysis of plus-strand RNA (Additional file 1). The EC_50 _values ranged from 12.3 to 21 μM, yet the level of cellular GAPDH RNA was not changed at these concentrations. The EC_50 _values were positively correlated with the IC_50 _values, suggesting there was little variation in the membrane permeability of each compound. In the presence of the compounds naïve Huh-7 cells showed an altered viability as measured by a standard MTT assay. The CC_50 _of G05 compound for naïve Huh-7 cells was 77 μM (Figure 1, a). The G05 compound was not a nucleoside analogue, suggesting that it may include a noncompetitive mechanism of inhibition. That was confirmed by measuring the [^32^P]-UMP incorporation by recombinant NS5B (C-terminal 21 amino-acids deleted form) in the presence of various concentrations of G05. The Km for UTP remained unchanged while the Vmax decreased when the concentration of G05 increased (Figure 1, b). The Lineweaver-Burk plot (Figure 1, c) suggests that the compound may interact with the HCV NS5B polymerase at a site other than the UTP binding site.

**Figure 2:**
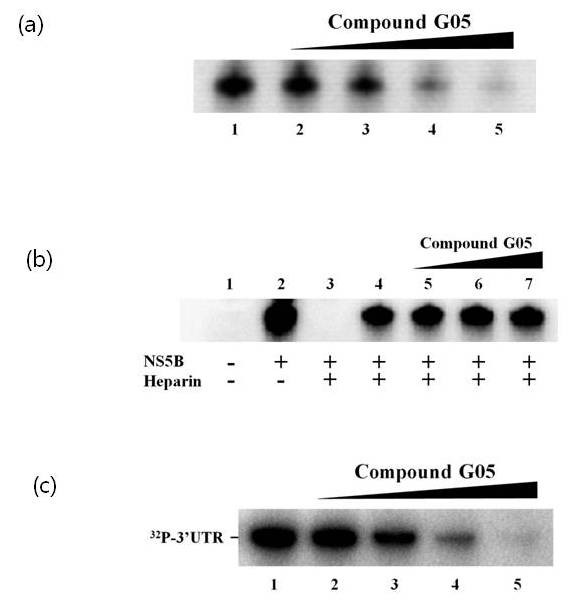
**G05 did not inhibit elongation step of RNA synthesis but inhibited RNA binding of the polymerase**. (a) The G05 compound reduced the amount of the newly synthesized RNA strand in a dose-dependent manner. The compound was added to a [^32^P]-UMP incorporation reaction using recombinant NS5B and poly(A)-oligo(dT) template at a concentration of 1 5 10 or 15 μM (lanes 2-5). (b) Single processive cycle conditions were set up with heparin an RNA polymerase trapper. Lane 1; RNA product in the absence of NS5B lane 2; RNA product in the presence of NS5B lane 3; RNA product in the presence of NS5B with the addition of heparin prior to the template; lane 4; single processive reaction without G05 compound lanes 5-7; single processive reaction at a concentration of 1 5 or 10 μM G05 compound respectively. (c) Inhibition of binding between recombinant NS5B and template RNA was measured. Recombinant hexahistidine-tagged NS5B was preincubated with G05 at various concentrations before adding 3' YTP RNA. After incubation the mixture was pulled down with Ni-NTA resin and the RNA was analyzed in a gel electrophoresis. Lane 1; no inhibitor lane 2; 0.5 μM G05 added lane 3; 1 μM G05 added lane 4; 5 μM G05 added lane 5; 10 μM G05 added.

As a noncompetitive inhibitor, G05 may either inhibit the initiation step or the elongation step of the polymerization reaction. We tested if the compound inhibited the initiation step of RNA synthesis using heparin. Heparin is a known polymerase inhibitor trapping free enzyme dissociated from the template [19] and was used to create a single processive reaction in this experiment. The NS5B and poly(A)-oligo(dT) template were mixed and preincubated at room temperature for 90 minutes before adding 2.5 μg of heparin, 10 μCi of α-[^32^-P]-UTP, and 50 μM UTP for the polymerization reaction. Thereafter, the G05 compound was added and the reaction mixture was further incubated and visualized after running on a polyacrylamide gel. Since the nucleotide mixture was added along with heparin, the level of RNA synthesis could only be measured from the preformed template-enzyme complex. In the presence of an increasing concentration of the G05 compound, the amount of newly synthesized RNA did not change (Figure 2, a), which suggests that the compound inhibited the initiation step of RNA synthesis rather than the elongation step. In the absence of heparin, the compound inhibited RNA synthesis in a dose-dependent manner (Figure 2, b). The inhibition mode was further supported by an initiation step assay. Inhibition of binding between recombinant NS5B and template RNA was measured as follows; purified recombinant NS5B was preincubated with G05 at various concentrations. In vitro transcribed 3' UTR RNA was added to each reaction and incubated before pulldown with Ni-NTA agarose beads (Qiagen, USA). In the presence of an increasing concentration of the compound, the binding of NS5B to the template RNA decreased dose-dependently (Figure 2, b), showing a direct inhibition in the initiation step.

The HCV NS5B polymerase is a well characterized enzyme and a druggable target based on the identification of at least three allosteric binding pockets in addition to the active site [20]. Accordingly, when screening a chemical library against HCV NS5B, we found a series of thiobarbituric acid compounds to be potent inhibitors of HCV NS5B polymerase. Based on the data presented in this study, the compound would appear to bind to an allosteric site in the enzyme and inhibit the initiation step of RNA synthesis in a noncompetitive manner. In addition to NS5B, the HCV replicase complex is also known to include other viral proteins, such as NS3, NS4A, and NS5A [21]. Plus, various cellular factors have also been suggested to be involved [22]. However, in the present results, G05 was found to be active against the purified recombinant NS5B in a biochemical enzyme assay, suggesting a direct interaction of the compound with the enzyme rather than an indirect influence due to interactions with cofactors. The compound was also active in a subgenomic replicon cell-based assay, meaning that it exerted the same effect in a cellular environment. They were able to pass through the cellular membrane and reach the perinuclear region where HCV replicase complex was reported to localize [23]. This study may provide some useful clues for development of antiviral therapy for hepatitis C virus.

## Competing interests

The authors declare that they have no competing interests.

## Authors' contributions

J-HL investigated the mechanism of action of the compound. SL and MYP contributed in the screening stage of the compound. HM conceived of the study, and participated in its design and coordination. All authors read and approved the final manuscript.

## Supplementary Material

Additional file 1 **Chemical structures and inhibitory effects of selected compounds**. * The IC_50 _was measured by a [^32^P]-UMP incorporation assay using poly(A)-oligo(dT) template and recombinant NS5B and represents the concentration of the inhibitor showing a 50% reduction in the recombinant NS5B polymerase activity. Unit = μM. # The EC_50 _was measured by real-time RT-PCR analysis and represents the concentration of the inhibitor showing 50% reduction in the RNA level in a Huh7 cell harboring the HCV subgenomic replicon. Unit = μM. Click here for file
