# Supplementary figures and images for: Characterization of thiobarbituric acid derivatives as inhibitors of hepatitis C virus NS5B polymerase
Source: Virol J. 2011 Jan 14;8:18. doi: 10.1186/1743-422X-8-18 (PMC3032711; doi:10.1186/1743-422X-8-18)

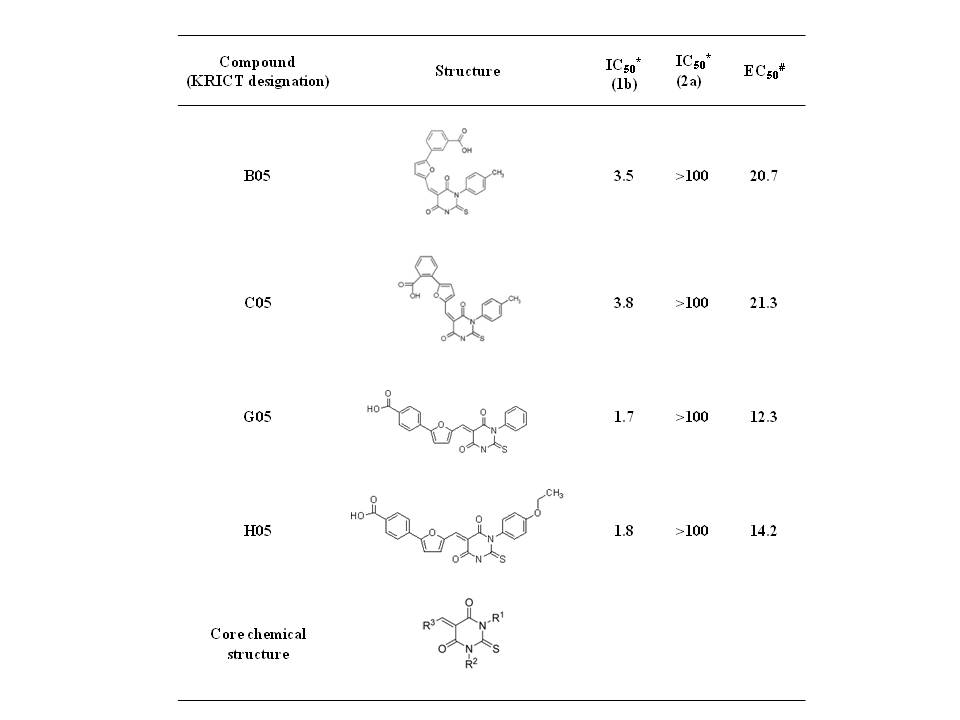

Supplement: Additional file 1 — Chemical structures and inhibitory effects of selected compounds. * The IC50 was measured by a [32P]-UMP incorporation assay using poly(A)-oligo(dT) template and recombinant NS5B and represents the concentration of the inhibitor showing a 50% reduction in the recombinant NS5B polymerase activity. Unit = μM. # The EC50 was measured by real-time RT-PCR analysis and represents the concentration of the inhibitor showing 50% reduction in the RNA level in a Huh7 cell harboring the HCV subgenomic replicon. Unit = μM. [file 1743-422X-8-18-S1.JPEG]
